# Compassion and the quality of life of the inpatient healthcare team

**DOI:** 10.1192/j.eurpsy.2024.1483

**Published:** 2024-08-27

**Authors:** A.-C. Bredicean, C. Giurgi-Oncu, L. Palaghian, D. Tabugan, A. Neagu, G. Covaci, R. Homeag, S. Ursoniu

**Affiliations:** ^1^ “Victor Babes” University of Medicine and Pharmacy; ^2^„Dr. Victor Popescu” Military Emergency Clinical Hospital, Timisoara, Romania

## Abstract

**Introduction:**

Nurse-patient relationships and interactions during inpatient care evoke feelings of empathy and compassion. Compassion can lead to satisfaction, but also to exhaustion. Compassion fatigue is a commonly used concept that signifies the exhaustion of healthcare personnel due to the specific activities and repeated exposure to the suffering of others. This manifests through physical and emotional over-tiredness, anxiety, anger and irritability, low vitality, social isolation, diminished sense of enjoyment of one’s career, cognitive disorders, and sleep disturbances.

**Objectives:**

To assess the level of compassion of the healthcare staff employed in a Romanian general hospital.

**Methods:**

The study sample included 256 nurses working in a general hospital. To identify socio-demographic data we applied a specific questionnaire, and subsequently we also used the PROQOL scale (Professional Quality of Life Scale). All data were statistically analysed.

**Results:**

The majority of healthcare professionals in our sample belong to the 40-49 age group (39.45%). Regarding work experience in the healthcare system, the majority(43%) have been working for over 10 years. 78.52% of nurses reported a high level of compassion satisfaction. Burnout was not identified in the majority of our sample (54.3%). We noted that the number of the staff affected by compassion fatigue increases proportionally with the years of work experience (P=0.033).

**Conclusions:**

A high level of compassion satisfaction in medical professionals leads to a remarkable improvement in the quality of the healthcare they are providing. However, our study results suggest that compassion fatigue tends to increase in line with the years of work in healthcare

**Disclosure of Interest:**

None Declared

